# Early diagnosis of oesophageal cancer

**DOI:** 10.1038/sj.bjc.6605126

**Published:** 2009-06-09

**Authors:** E L Bird-Lieberman, R C Fitzgerald

**Affiliations:** 1 MRC Cancer Cell Unit, Hutchison-MRC Research Centre, Hills Road, Cambridge CB22 0XZ, UK

**Keywords:** squamous cell carcinoma, adenocarcinoma, Barrett's oesophagus, biomarkers, inflammation, cell cycle, endoscopy

## Abstract

Squamous cell carcinoma and adenocarcinoma of the oesophagus are cancers that develop from distinct epithelial sub-types; however, they are both related to chronic inflammation of differing aetiologies. Inflammation leads to somatically inherited genetic mutations altering control of the cell cycle, DNA replication and apoptosis, which together result in autonomous and uncontrolled proliferation. These cancers have often metastasised to lymph nodes and distant organs before symptomatic presentation and therefore carry a poor prognosis. It is therefore vital to diagnose oesophageal cancer at an early stage, before the development of symptoms, when treatment can dramatically improve prognosis. Understanding the pathogenesis of these cancers is vital to guide early diagnostic strategies.

Cancer of the oesophagus presents symptomatically late in the course of the disease and, despite currently available therapies, carries a poor prognosis. The annual incidence of oesophageal cancer in the United Kingdom is 9.6 out of 100 000 population and it has a very similar annual mortality of 8.7 out of 100 000 population because of the overall poor 5-year survival rate of under 10% (CRUK, 2009). Squamous cell carcinoma (SCC) and adenocarcinoma (AC) are the two main histological types of oesophageal cancer that will be discussed, although rarities, such as adenoid cystic, mucoepidermoid, adenosquamous, small cell carcinoma, sarcoma, melanoma and lymphoma, can occur.

For the purpose of this review ‘early’ oesophageal cancer will be defined as high-grade dysplasia, or tumour limited to the mucosa (T_*in situ*_) or submucosa (T_1_) and not extending into the muscular wall of the oesophagus.

The stage (classified according to the T, N, M system) at which oesophageal cancer is detected is the most important factor in determining prognosis. The rate of lymph node metastasis in both SCC and AC is related to the depth of invasion and neither tend to be associated with lymph node metastasis when invasion is limited to the muscularis mucosa (Endo and Kawano, 1997; Ancona *et al*, 2008). The majority of oesophageal cancers that present with symptoms have invaded to the muscularis propria (T_3_) and have already spread to local lymph nodes (N_1_); this is the reason for the poor prognosis.

In stark contrast to oesophageal cancers that present with symptoms, the 5-year survival for early disease is over 90% (Wang *et al*, 2009). The survival benefit is because treatments are more effective at this early stage. These statistics should, however, be interpreted with an element of caution because of the phenomenon of lead time bias. When screening or surveillance detects a pre-symptomatic cancer, the survival from this cancer will appear longer than if it was diagnosed after the appearance of symptoms at a later date, even if early intervention provided no benefit.

Historically, radical oesophagectomy was necessary to treat early oesophageal cancer. Endoscopic therapeutic techniques have, however, advanced rapidly in the last decade and can now be used as an alternative for the treatment of early cancer (Singh and Sharma, 2009). Endoscopic therapy can take the form of a dissection of the superficial layers of the oesophageal lumen (endoscopic mucosal or sub-mucosal resection) or generalised mucosal ablation (e.g., radiofrequency ablation, photodynamic therapy or argon plasma coagulation). Surgical techniques have also evolved and laparoscopically assisted oesophagectomies are being undertaken for early disease.

## Epidemiology

### Squamous cell oesophageal cancer

Squamous cell carcinoma accounts for the bulk of oesophageal cancer worldwide, particularly in the developing world, and has great geographical as well as sociocultural variation in incidence. Some of the highest SCC rates are in northern China and northern Iran where the incidence is 1 out of 1000. In the United States, SCC rates fell by 3.6% each year between 1998 and 2002; this decrease occurred in most ethnic groups and in both sexes (Trivers *et al*, 2008). This decrease in incidence is thought to be because of a reduction in smoking.

### Oesophageal adenocarcinoma

Adenocarcinoma accounts for up to 50% of oesophageal cancers in the West, particularly in the male Caucasian population (Parkin *et al*, 2002), in contrast to the prevalence of SCC in the developing countries. In Britain, age-standardised incidence rates of AC have risen by just under 40% every 5 years (Lepage *et al*, 2008), and have recently been highlighted by the Chief Medical Officer as a ‘serious pathological concern’.

## Clinical stages of disease

### Symptomatic presentation

The expansible nature of the oesophagus means that early cancers rarely present symptomatically because circumferential involvement, or considerable penetration into the lumen, is required to cause dysphagia, and invasion into adjacent structures is required to trigger pain. The diagnosis of early oesophageal cancer should therefore not be focused at this late stage of the pathway, but rather at asymptomatic individuals.

The detection of oesophageal cancer before the development of symptoms requires screening and/or surveillance. In the West, the preponderance of AC means that research into screening and surveillance of oesophageal cancer has been focused on AC rather than SCC.

Surveillance is the repeated application of a test (such as, endoscopy) that allows detection of disease at a stage when intervention may improve outcome. This process is made more cost effective by limiting it to those at particular risk, for example, those with Barrett's epithelium (BE) (+/−dysplasia) who are at increased risk of AC (Gerson *et al*, 2004). We would expect to increase detection of early cancers if screening is used in addition to surveillance, although if this was dependent on endoscopy, the cost effectiveness may in turn be decreased. Screening is the one-time application of the same test to allow detection of early cancers and those at risk within the general population.

### Surveillance for adenocarcinoma

In the United Kingdom, the current evidence for the overall benefit of oesophageal surveillance programmes for AC is limited and they are not universally recommended (http://www.bsg.org.uk, 2005). However, in the absence of useful alternatives, patients with known BE are usually offered the choice of entry into a surveillance programme, which consists of endoscopic inspection and multiple biopsies (taken from macroscopically abnormal areas and quadrantically every 2 cm from areas, which have no macroscopic features of cancer) every 2–3 years. The only biomarker currently used to guide endoscopic or surgical intervention is high-grade dysplasia found within these biopsies, but patches of dysplasia may be missed despite extensive biopsying and thus false reassurance may be provided. It is estimated that overall 0.6% of people with Barrett's oesophagus will progress to AC each year (Yousef *et al*, 2008). As a result of this concern, many patients at very low risk continue to undergo endoscopic surveillance.

### Screening for adenocarcinoma

Currently Barrett's oesophagus and squamous dysplasia are diagnosed by endoscopy and biopsy. The associated balance of risk, benefit, cost and psychological burden has not justified the implementation of a national screening programme (Gerson *et al*, 2004). Barrett's oesophagus is therefore generally diagnosed as part of a general investigation for reflux or dyspepsia, or incidentally during endoscopy for an alternate indication. New, less invasive and cheaper screening tools are being sought; and most current gastroenterology infrastructures would not be able to support the introduction of an endoscopic screening program.

### Screening for squamous cell cancer

Cytological screening techniques have been developed for the early detection of SCC and have been used particularly in the Linxian region of China, where there are large numbers of people who are asymptomatic, but at high risk of SCC. A deflated balloon, covered with netting, is swallowed, inflated when in the stomach, and retracted along the oesophagus. Attached cells are smeared onto slides, stained with Papanicolaou's stain and examined (Dawsey *et al*, 1997). This cytological diagnosis has low sensitivity (14–36%) for the detection of cancers, which have already been detected on biopsy (Dawsey *et al*, 1997) and endoscopic screening is taking place in preference.

## Endoscopic techniques available for early diagnosis

Traditional white-light endoscopy can identify macroscopic features of early cancer, such as nodules, ulcers or strictures. Unfortunately, early cancers, particularly at the high-grade dysplasia stage, often appear macroscopically normal. Given the Western preponderance of AC, newer endoscopic imaging techniques have been focused in this area and are reviewed below. Many of the newer techniques are plagued by the need for expert histological knowledge to interpret images; they thus depend on operator experience and may not be applicable to the general endoscopic setting. These techniques are also time consuming and rely on consistent operator technique and interpretation.

### Chromoendoscopy

Chromoendoscopy is the application of a chemical capable of selective light absorption, which results in colouration of certain organic compounds to enhance endoscopic visualisation. The application of Lugol's iodine has been shown to improve visualisation of SCC and its pre-cancerous stage (Dawsey *et al*, 1998). Methylene blue has also been used to improve detection of dysplasia in Barrett's oesophagus, but subjectivity has meant results are difficult to reproduce (Ormeci *et al*, 2008), and there has been some concern over potential carcinogenicity of methylene blue (NTP, 2008).

### Trimodal imaging

A trimodal endoscope, which combines the ability to provide high-resolution white-light endoscopy, autofluorescence and narrow-band imaging, is now commercially available. Autofluorescence is used in an attempt to avoid many of the problems associated with administration of exogenous chromophores. There is limited randomised evidence to show improvement of detection of high-grade dysplasia over white light alone (Kara *et al*, 2005), and it is particularly troubled by false positives. Narrow-band imaging aims to highlight the vasculature and mucosal pit pattern by filtering out longer wavelengths of light. Some studies have shown some improvement in the detection of dysplasia compared with white-light endoscopy (Wolfsen *et al*, 2008). Areas of concern, which seem to be more purple in colour on autofluorescence can then be examined with narrow-band imaging, with the aim of decreasing the false-positive rate (Kara *et al*, 2006). Some clinical trials comparing trimodal imaging with white-light endoscopy and random quadrantic biopsies have shown an increased detection of dysplasia and early oesophageal cancer (Curvers *et al*, 2008); however, this is not universally the case (Kara *et al*, 2006).

### Confocal fluorescence microscopy

Confocal fluorescence microscopy attempts to highlight morphological changes in tissue, which occur in dysplasia, through imaging of the autofluorescence properties of the mucosa. Excitation with blue light leads to emission of longer wavelengths of light from molecules, such as haemoglobin or collagen. The administration of intravenous fluorophores (e.g., fluorescein) can improve depth of visualisation compared with using autofluorescence alone.

Confocal fluorescence microscopy aims to provide an image consistent with the histology of the tissue being examined in an *in vivo* setting, but so far falls short of this aim. It was found to achieve a high-negative predictive value (99%) *in vivo*, but sensitivity was poor (positive predictive value 44%) (Pohl *et al*, 2008).

### Elastic scattering spectroscopy

The epithelial elastic scattering index depends on the composition of sub-cellular components, such as the nucleus and mitochondria, which alter during malignant transformation. An optical probe can be inserted through the instrument channel of the endoscope to give a measurement, which eliminates the problems of observer variability. This technique has been shown to have a high sensitivity (92%), but poor specificity (60%) (Lovat *et al*, 2006). However, prospective validation is awaited and elastic scattering spectroscopy does not circumvent the problem of sampling error, as it does not allow sampling of the entire segment.

### Optical coherence tomography

Optical coherence tomography measures the backscattering of infrared light to produce a high-resolution image of the epithelium in cross section (up to 3 mm in depth), in a similar way to ultrasound imaging. *In vitro* and *ex vivo* feasibility studies have been promising, but *in vivo* studies showing adequate sensitivity and specificity for detection of dysplasia are lacking at present (Testoni and Mangiavillano, 2008).

## Molecular pathogenesis of oesophageal cancer

**Figure 1:**
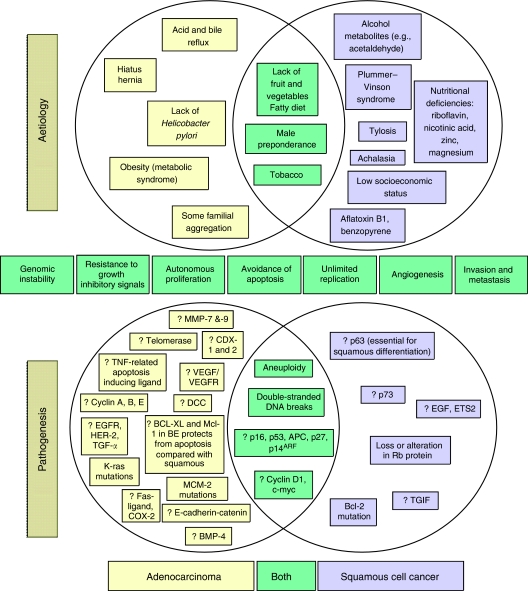
The common and distinct known aetiology and pathogenesis of oesophageal adenocarcinoma (AC) and squamous cell carcinoma (SCC). Adenocarcinoma and SCC develop the characteristics of cancer through somatic mutations, some of which are shown above. The areas in which AC and SCC overlap are shown in green.

Both AC and SCC are thought to develop through a series of somatic mutations or epigenetic changes. These genetic changes allow six of the clinical characteristics of cancer to occur: resistance to growth-inhibitory signals; autonomous proliferation; avoidance of apoptosis; unlimited replication; angiogenesis; and invasion and metastasis (Hanahan and Weinberg, 2000). Many of the mutations, which lead to these characteristics, will confer a survival benefit on the mutated cells, and lead to clonal expansion at the expense of other cells. Figure 1 shows the changes that have been shown to occur in the pathogenesis of AC and SCC; many of these changes have been suggested to be suitable biomarkers for the development of cancer.

Chronic inflammation is thought to be the precipitating factor in both SCC and AC. In SCC, the chronic inflammation is thought to be precipitated by toxins, including cigarettes, alcohol and aflatoxin B1 (from mouldy food). In AC, the inflammation is thought to be triggered by acid and bile reflux into the oesophagus as this is the strongest risk factor for the development of the columnar metaplasia known as BE (Conio *et al*, 2002). Barrett's epithelium increases the chance of developing AC to 0.5% per patient per year (Shaheen *et al*, 2000) and it is thought that on-going reflux and other factors, such as dietary nitrates contribute to progression to cancer.

### Molecular techniques that can be used on oesophageal tissue

An ideal technique to determine high risk of progression to cancer would not only be sensitive and specific, but would also be quick, non-invasive and would not require specialist interpretation. Endoscopic biopsy surveillance does not meet this ideal on many levels. It is invasive and expensive, biopsy taking is laborious and samples only a small area of the heterogeneous epithelium and there is inter-observer variation in the time-consuming pathological interpretation. However, multiple biopsies, which have been fixed in formalin, do allow immunohistochemistry to be performed. Immunohistochemistry can inform about the presence of certain biomarkers, their specific location within the tissue and provide some quantitative information; immunohistochemistry for MCM-2 and cyclin-A have been used, for example, to predict risk of progression to AC (Lao-Sirieix *et al*, 2007).

Cytological sampling allows a more rapid sampling of a larger surface of superficial epithelium compared with biopsies, but the sampling is less specific because of contamination with other cells and has been attempted in both AC and SCC. Harvesting cells for cytological analysis can be performed at endoscopy using a brush inserted through the instrument channel; however, it would be preferable to obtain specimens in a less invasive manner. The mesh-covered balloon method for obtaining cytology has already been discussed.

### Cytology for detection of squamous cell cancer

Cytological samples obtained by this method have been evaluated for the presence of methylation of eight genes, which have been shown to be methylated in SCC using quantitative methylation-specific PCR. The best sensitivity and specificity was obtained when a combination of four of these genes was used (AHRR, p16INK4a, MT1G and CLDN3). However, they were not good enough markers to be of clinical use as the sensitivity and specificity were only 50 and 68%, respectively (Adams *et al*, 2008).

### Cytology for screening for Barrett's oesophagus

The capsule sponge is another method that has been developed for obtaining cytological specimens in a minimally invasive manner. The sponge is contained within a gelatine capsule, which dissolves when it comes into contact with gastric contents. The sponge is thus liberated and can then be withdrawn on a thread through the mouth; this can be performed in a primary care setting. It is being used to investigate molecular markers, MCM-2 and TFF3, in screening for BE, but could also be used in surveillance if appropriate biomarkers were available.

### Automation of cytological analysis

Ideally, analysis of cytological samples could be automated to minimise the need for time-consuming, operator-dependent cytological analysis. Flow cytometry, fluorescent *in situ* hybridisation (FISH) and PCR are techniques for which automated systems have been developed for the prediction of progression to AC.

Flow cytometry can be used to determine the percentage of cells with tetraploidy, which is associated with progression to cancer (Chao *et al*, 2008). The combination of a 4N fraction >6% with aneuploidy (an independent risk variable) improved the predictive power of flow cytometry. Unfortunately, flow cytometry requires a larger number of cells than is obtained with brushings and needs to be performed on biopsies, which sample only a small area.

Fluorescent *in situ* hybridisation makes use of fluorescently labelled DNA probes to detect chromosomal changes, such as aneuploidy, loss of tumour suppressor genes (e.g., p53 or p16) or oncogene amplification (e.g., Her-2). Despite the availability of automated FISH systems, the price and need for specialist analysis prevent its routine use in a surveillance setting; however, automated systems may improve in the future. Amplification of c-myc, EGFR and the 20q12 loci have been proposed as prognostic markers for the development of dysplasia/AC and can be detected using FISH on brush cytology specimens (Rygiel *et al*, 2008). It should be remembered that FISH will not detect point mutations or hypermethylation.

Quantitative methylation-specific PCR has been used to determine methylation status and levels of the genes p16, HPP1 and RUNX3. The variations in methylation of these genes have been used, in combination with clinical data, to estimate risk of progression to early cancer in Barrett's oesophagus (Sato *et al*, 2008). These markers have progressed to retrospective studies to define the criteria for a positive screening test.

### Serum biomarkers

Identification of biomarkers for oesophageal cancer in serum is attractive because of the ease of obtaining samples for analysis and would be an ideal method for screening or surveillance in a primary care setting. Tumours release their proteins into the circulation when cells die and through active secretion. They also cause systemic changes in cytokines and growth factors, and alterations in the immunological profile because of cancer antigenicity. Antibodies to the altered cancer glycome, such as the antibody to α-fetoprotein (AFP), are already used in the diagnosis of other cancers. An individual serum biomarker for oesophageal cancer with sufficient sensitivity has yet to be identified. As our understanding of the changes that take place in the development of oesophageal cancer improve, more biomarkers are likely to be identified. Given the limited sensitivity and specificity of biomarkers, which have thus far been identified, it is most likely that a panel of serum biomarkers will prove optimal. Biomarker array systems are being developed that could automatically detect the multiple markers of interest. In a small study of one such array, Fas ligand was shown to have a sensitivity of 83% and a specificity of 100% for oesophageal AC (Kilic *et al*, 2008).

Blood might also be screened for tiny amounts of tumour-specific DNA, for example, using methylation-specific PCR to look for hypermethylation of the p16 promoter in SCC (Hibi *et al*, 2001) or reverse-transcription PCR to look for ΔNp63 expression in peripheral blood in SCC (Koike *et al*, 2002). Reverse-transcription PCR has shown that expression of squamous cell carcinoma antigen 2 (SCCA2) mRNA in peripheral blood correlates well with expression in the cancer itself and with levels of SCCA2 in peripheral blood detected by ELISA. However, although blood levels of SCCA2 mRNA increased along the pathway to cancer, this test was not sufficiently sensitive to be used alone as a biomarker (Yang *et al*, 2008).

## Conclusion

Oesophageal cancer presents late and carries a grave prognosis. It is hoped that understanding its pathogenesis will reveal biomarkers that can be used to diagnose these cancers early in order to improve outcome. Pre-cancerous changes are often associated with a heterogeneous field change. Single biomarkers identified thus far lack sufficient sensitivity and specificity and it is likely that multiple markers will need to be employed simultaneously. This biomarker profile approach may assist us not only in the early diagnosis of the condition, but also guide management.

It is possible that the new endoscopic techniques in development will allow us to image changes at the molecular level and allow detection of biomarkers associated with high risk of progression to cancer. Such a diagnostic endoscopic technique could be combined with immediate endoscopic therapy to eradicate mucosal areas of concern and prevent cancer development.
